# Soft Release Translocation of Texas Horned Lizards (*Phrynosoma cornutum*) on an Urban Military Installation in Oklahoma, United States

**DOI:** 10.3390/ani10081358

**Published:** 2020-08-06

**Authors:** Brett DeGregorio, Raymond Moody, Hannah Myers

**Affiliations:** 1 United States Geological Survey, Fish and Wildlife Cooperative Research Unit, University of Arkansas, 850 W Dickson St., Fayetteville, AR 72701, USA; 2 USAF 72nd ABW/CE, Environmental Compliance Natural Resources, Tinker AFB, OK 73145, USA; raymond.moody@us.af.mil (R.M.); hgeorge9093@gmail.com (H.M.)

**Keywords:** conservation, hard release, juveniles, *Phrynosoma*, soft release, translocation

## Abstract

**Simple Summary:**

Wildlife are moved (translocated) for numerous reasons, whether it is to establish a new population for conservation purposes or to move animals out of harm’s way. Unfortunately, animals that are moved often fail to survive in their new homes. Soft release is a technique of penning animals at the release site before letting them free, which allows them to become acclimated to their new surroundings and prevents them from immediately trying to return to their capture location. We soft-released Texas horned lizards at an urban military installation in Oklahoma, United States. While soft-released adults did have reduced post-release movements, they also had low survival, suggesting that they do not respond well to translocation. However, post-release survival of juveniles was high and equivalent to resident juveniles. Translocation efforts of Texas horned lizards may be most successful if they focus on relocation of juveniles rather than adults.

**Abstract:**

Wildlife translocation is an often-used technique to augment populations or remove animals from harm’s way. Unfortunately, many translocation efforts fail to meet their goals for myriad reasons, particularly because translocated animals make large, erratic movements after release, which can result in high mortality rates. Soft release, holding animals in acclimation pens for some period of time at the recipient site before release, has been proposed as a technique to reduce these large movements and increase the survival of translocated animals. Here, we compared the survival and movement patterns of soft-released Texas horned lizards (*Phrynosoma cornutum*) with resident lizards, as well as hard-released lizards from a prior study. Juvenile lizards that were soft-released had high survival rates similar to resident lizards, despite still moving more frequently and occupying larger home ranges than residents. Conversely, soft-released adult lizards had survival rates similar to those that were hard-released, and much lower rates than resident adults. Curiously, soft-released adults did not have significantly higher movement rates or home range sizes than residents. Our results suggest that caution should be used before adult Texas horned lizards are translocated. However, juveniles responded well to soft release, and future research should explore whether they are more resilient to translocation in general, or if soft release provided a specific survival advantage. Contrary to our predictions, the survival of translocated animals was not related to their post-release movement patterns, and the mechanism underlying the observed survival patterns is unclear.

## 1. Introduction

As wildlife populations decline and their habitat is encroached upon by humans, land managers are adopting techniques like wildlife translocation to maintain or augment viable populations [1]. Translocation is the intentional release of captive-propagated or wild-caught animals into the wild for the purpose of establishing a new population, augmenting a critically small population, or managing animals that are in harm’s way [2,3]. Despite substantial investments of time, energy, and resources, these endeavors often fail to establish wild populations (33–52% failure rate [1]), yet the practice is becoming more common on private, state, and federal lands [3,4]. Furthermore, many translocation endeavors fail to define criteria for success by which the effectiveness of translocation projects can be objectively assessed [3,5,6].

The default translocation approach involves simply releasing animals unrestrained into the new environment (i.e., hard release). In general, mortality rates for animals translocated in this manner are high [4,7]. Hard-released animals are typically disoriented, and make long, erratic movements that expose them to predators or vehicles [8,9,10]; this may decrease the time available for activities like foraging and mating. If animals were translocated short distances, their likelihood of returning to their point of capture (i.e., homing) can be quite high [11], which may return them to harm’s way. A valuable alternative to hard release that potentially reduces these deleterious impacts is soft release.

Soft release entails placing translocated animals in outdoor enclosures at the release site prior to full release, allowing animals to experience local environmental conditions, develop fidelity to a site, and reduce the urge to immediately attempt to home [12]. Many translocated animals immediately attempt to return to their site of capture, and animals confined in soft release pens often display increased movement around the periphery of the pen for some period of time; it has been suggested that releasing animals from pens after they cease this behavior might be beneficial [12]. Soft release often allows animals to develop, practice, and display natural behaviors, such as foraging, mating, thermoregulating, and burrowing, and has proven effective for a number of successful translocation projects [13,14,15,16] including, recently, for the Texas horned lizard (*Phrynosoma cornutum*; [17]). For example, gopher tortoises (*Gopherus polyphemus*) that were soft-released in South Carolina moved shorter distances and experienced higher survival upon release than individuals that were hard-released [13]. Soft release is a promising, low-risk approach for decreasing post-release movement and thus increasing the survival of translocated animals. However, more empirical studies are needed before soft release can be widely adopted [7].

Only a small handful of translocation efforts have applied soft release to lizard species. Knox and Monks [15] and Knox et al. [18] showed that soft releasing Jewelled Geckos (*Naultinus gemmeus*) resulted in less dispersal behavior and smaller home ranges than hard released individuals. Fitzgerald et al. [19] attributed the successful reintroduction of St. Croix Ground Lizards (*Ameiva polops*) to Buck Island at least partially to soft release. However, short-duration (1–5 days) soft release may cause stress to lizards and make them more likely to disperse after release [20]. Our objectives here were to assess how a 2-week soft release period affected the survival and movement patterns of translocated Texas horned lizards (*Phrynosoma cornutum*) on a military installation in Oklahoma, United States.

Texas horned lizards have declined throughout much of their native range [21], primarily due to anthropogenic habitat alteration and destruction, as well as the use of pesticides on ants, their main food source, and the introduction of the invasive red imported fire ant (*Solenopsis invicta*: [21,22]). The species is currently designated as a Tier 1 species of greatest conservation need in Oklahoma, and is listed as state threatened in the neighboring state of Texas. On Tinker Air Force Base construction of housing and industrial infrastructure has caused the destruction of Texas horned lizard habitats. Installation biologists have attempted to mitigate the harm by translocating lizards from construction areas to suitable, protected areas of the installation. However, prior research on Tinker Air Force Base has shown that 17 hard-released adult lizards generally had lower survival (annual survival of 16%, 95% CI: 0.00–0.34) compared to resident lizards (57%, 95% CI: 0.21–0.93) at the same site [23] Furthermore, mortality for hard-released lizards often happened quickly, with 11 of 17 lizards dying or disappearing within 2 months, and 14 of 17 dying or disappearing within 6 months [23]. Translocated lizards also had larger home ranges than resident lizards [23]. Thus, hard release appears to be an unsuitable conservation approach for Texas horned lizards at this site, and an alternative solution is needed. Our goals here were to test if soft release could improve the survival and reduce the post-release movement of translocated Texas horned lizards on Tinker Air Force Base. Specifically, we tested if translocated lizards soft released for two weeks at the release site would have reduced movement rates, space use, and higher survival relative to hard-released lizards and comparable to resident lizards. Furthermore, we tested the effects of soft release on both adult and juvenile Texas horned lizards, because these age classes have been shown to have very different movement patterns and survival rates [22,23,24,25], and thus might respond to translocation differently.

## 2. Materials and Methods

### 2.1. Study Site

**Figure 1:**
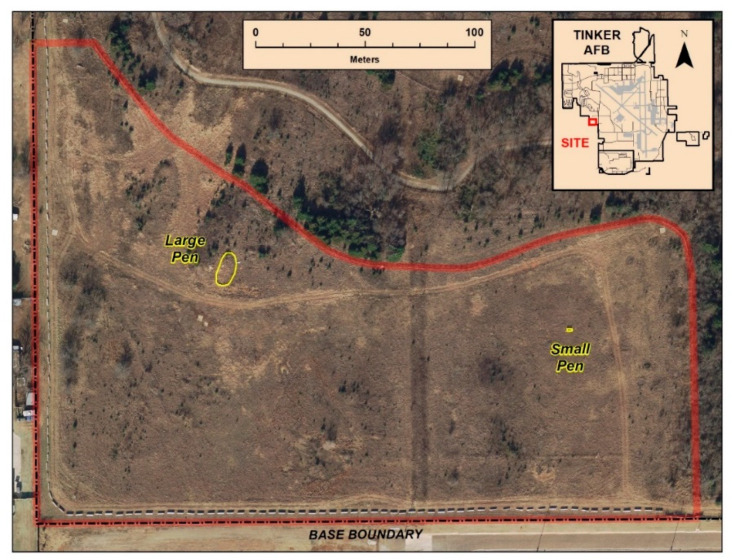
Translocation site on Tinker Air Force Base, Oklahoma. Two soft-release translocation pens were constructed in this 4 ha grassland.

Tinker Air Force Base (TAFB) covers approximately 2000 ha in Oklahoma County, Oklahoma (35°24′58″ N, 97°24′41″ W). It is located in the southeastern quadrant of greater metropolitan Oklahoma City. The installation contains approximately 500 ha of natural habitat that are managed and protected for wildlife and recreation. These natural areas are interspersed amongst urban development, housing developments, and roads; thus, connectivity between natural areas is typically low. Much of the previous work studying Texas horned lizards on TAFB [23,24,25,26] have focused on an area called Wildlife Reserve 3, an approximately 15 ha grassland located within a larger urban greenway (ca. 183 ha). Starting in 2016, we expanded our study to include the “Translocation Site” (Figure 1). The Translocation Site is an approximately 4 ha grassland known to currently have resident lizards and identified as suitable Texas horned lizard habitat by a site-wide habitat suitability model [23]. The translocation site has deep, well-drained clay soil and is covered in prairie vegetation comprised of big bluestem (*Andropogon gerardii*), little bluestem (*Schizachyrium scoparium),* plains bluestem (*Bothriochloa ischaemum*), indiangrass (*Sorghastrum nutans*), side oats grama (*Bouteloua curtipendula*), Maximilian sunflower (*Helianthus maximiliani*), tall fescue (*Lolium pratense*), and eastern redcedar (*Juniperus virginiana*). We chose this site as a specific release site for soft release translocated lizards so that the release of additional lizards would not interfere with the long-term studies occurring on Wildlife Reserve 3. While we knew resident lizards were present on the Translocation Site, we did not know how dense the population was. The lizards at the Translocation Site were naturally present there, and were not released as part of previous translocation efforts.

### 2.2. Study Animals and Radio Telemetry

For this study, we monitored the survival and movement of two groups of lizards: soft-release translocated lizards and resident control lizards. Because Texas horned lizards have previously been hard-released at this site, and the survival of translocated lizards was low [23], we did not hard-release any additional lizards as part of this study; however, we do qualitatively compare our results to those from the previous effort. We found candidates for soft release by surveying for lizards in urban areas adjacent to prairie habitats. These areas consisted of marginal habitat surrounding or interspersed with parking lots, buildings, and other infrastructure on the installation. We surveyed these areas approximately three times per week, and would respond to calls from the public who located lizards in these areas. Often lizards dispersing from suitable prairie habitat find themselves in such habitat and are in danger of mortality from vehicles, desiccation and overheating, or predators. Historically, lizards found in these areas would be hard-released at the nearest suitable habitat patch or translocated to Wildlife Reserve 3. Starting in 2016, we would soft-release these lizards at the Translocation Site. Additionally, we surveyed suitable prairie habitat on the installation where construction activities were scheduled to take place in the future, due to expansion of aircraft infrastructure. Because these lizards were presumed to be in danger when their habitat was to be converted in the future, we deemed these individuals as good candidates for translocation and soft release. None of these lizards would have been familiar with the Translocation Site because of the distance between their capture sites and the urban matrix separating the Translocation Site from these capture locations. We surveyed these pre-construction areas approximately three times per week early in the field season, and then less frequently as the season progressed and we needed to spend more time monitoring already translocated lizards. All but one translocated lizard came from Tinker AFB, and most lizards were moved between 2 and 20 km to the release site.

Resident lizards were found by visually surveying suitable habitat at the Translocation Site or opportunistically during monitoring and tracking activities. Visual searches consisted of slowly walking back and forth across suitable habitat while looking for lizards. We recorded morphometric information for each captured lizard, including snout-vent length (SVL), total length (TL), mass, and sex. Additionally, we individually marked each lizard >5 g in mass with a passive integrated transponder (PIT) tag (0.5 g). Lizards too small to receive PIT tags were marked by toe clipping.

We dorsally attached radio transmitters (model BD-2, 0.95–1.95 g; Holohil Systems Ltd., Carp, ON, Canada) to adult lizards (85–93 mm, 14–20 g) using silicone adhesive and small elastic collars placed around the neck (total encumbrance was ≤10% of an individual’s mass). To track juveniles and hatchlings (<74 mm, <12 g), we glued harmonic radar diodes (low-barrier-height Schottkey barrier diodes that weighed only 1 to 12 mg) to their backs, and relocated them using handheld RECCO transmitter/receivers (RECCO Rescue Systems, Lidingo, Sweden). We tracked lizards between 3–5 times per week during the active season (April–November). Each time we located a lizard, we recorded its location using hand-held Trimble GPS Pathfinder Pocket Receivers (Trimble GeoXT, Terrasync 2.3, Strategic Consulting International, Oklahoma City, OK, USA) and stored location data in a geodatabase. Locations were recorded in universal transverse Mercator coordinates. We varied the times during which we tracked lizards each day, in order to avoid bias in behavior. In addition to recording the lizard’s location, we made notes about its behavior, habitat use, and meteorological conditions.

### 2.3. Soft Release

**Figure 2:**
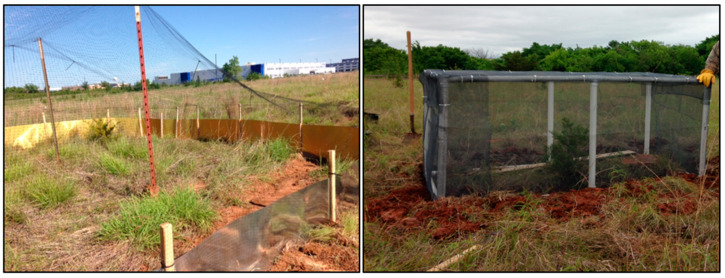
Soft-release pens built on Tinker Air Force Base, Oklahoma in 2016 to soft-release translocated Texas horned lizards (*Phrynosoma cornutum*). The pen on the left was constructed of aluminum flashing and wildlife netting and was approximately 0.01 ha in size while the pen on the right was constructed of PVC piping and fiberglass screen and measured 2 m long × 1 m wide × 1 m tall.

In May 2016, we constructed two soft-release pens at the translocation site (Figure 1). The first pen was approximately 0.01 ha in size and had walls of aluminum flashing (Figure 2). The walls were constructed of 50.8 cm tall aluminum flashing, trenched approximately 8–10 cm into the ground and held upright by wooden stakes spaced approximately 2–3 m apart. The habitat within the pen was natural prairie vegetation interspersed with bare ground. We mounded sand to ensure that bare ground would be available for thermoregulation, as well as friable soil into which lizards could burrow. After one of the first lizards was removed from the pen, presumably by an avian predator, we used wildlife netting to create a “roof” over the pen to prevent predators from accessing the enclosed lizards. The second and smaller pen had a frame of PVC piping and was entirely enclosed with a fiberglass screen to prevent escape and to prevent any access by predators. The dimensions of the pen were 2 m long × 1 m wide × 1 m tall. The inside of the pen was a natural substrate with live vegetation, as well as a small water bowl sunk into the ground and a piece of wooden debris to provide additional cover (Figure 2). Lizards were held in the pens for two weeks before being manually released by hand just outside of the pens. We never had more than three lizards in any given pen at a time. Our general approach was to alternate use of the pens so that we had similar sample sizes of released lizards from each pen. However, during the spring of 2017, the larger pen needed extensive repairs from storm damage, and the smaller pen was used more frequently. Because lizards for translocation were found opportunistically, we never had groups of lizards being released at once, but rather releases happened throughout the year after individual lizards were found and had spent the 2 weeks in their pen.

### 2.4. Data Analysis

To analyze and compare the survival rates of soft-released and resident lizards, we calculated daily and annual survival rates using the Mayfield estimator. The Mayfield survival estimator assigns a fate to each lizard on each tracking event: 1 (survived) or 0 (died). This Mayfield method allows the staggered entry of individuals and assumes all individuals have the same probability of detection and of survival. We then used the number of failures (deaths) during the number of summed tracking days for all lizards. When a lizard went missing, we defined this as a failure, unless the transmitter battery life was nearing its estimated completion. Our conservative approach in assigning fate lead us to estimate survival rates as lower than what they likely were. We used an Akaike information criterion for small sample sizes (AICc) approach to rank candidate models assessing the differing impacts of various factors on the survival rates of the lizards. We evaluated a model for treatment that categorized each lizard as either a resident or soft-released. We also evaluated a model for the release pen to account for potential differences in survival associated with lizards being released from the small pen or the large pen. We also evaluated models for a year to account for variation in survival associated with differing environmental conditions between years and the day of year to account for uneven survival across the active season. Because annual survival rates of adults are naturally higher than for juveniles [25], we analyzed both age classes separately. We considered adults as lizards that had survived their first entire active season post-hatching, and juveniles as any lizard hatched that season or the previous season.

For each lizard, we calculated two metrics of movement to compare between resident and soft-released lizards. First, we calculated movement rate or average distance moved per day. We calculated this by recording the Euclidean distance between successive tracking locations and dividing by the number of days between tracking events. We then averaged these values for each individual across the entirety of the active season. Because of our uneven sample sizes, unequal variances, and non-normally distributed data, we used nonparametric tests to make comparisons between the movement rates of soft-released and resident lizards. Specifically, we used Kruskal–Wallis one way analysis of variance tests (PROC Nonpar1way) using SAS 9.4 (SAS Institute, Cary, NC, USA). Because juveniles and adults have been reported to have different patterns in movement and space use, we compared adults and juveniles separately.

In addition to movement rate, we calculated home range sizes for each lizard tracked during the study. We limited this analysis only to lizards tracked for more than 16 days. We calculated 95% minimum convex polygons (MCPs) for each soft-released and each resident lizard. We used the package “adehabitatHR” [27] in Program R v.3.4.3 (R Core Team 2017) to estimate the home ranges of individual lizards. Although kernel density estimators may provide a more sophisticated index of space use for wildlife, we chose the more simplistic MCP approach to facilitate comparisons with other studies and with prior studies conducted on TAFB.

## 3. Results

### 3.1. Survival

From May 2016–October 2018, we tracked 17 resident adult Texas horned lizard (THLs) (7 males and 10 females), for a total of 1317 tracking days (Table 1). Three had confirmed mortality events, and the others survived or were censored (transmitters fell off or the battery expired). One was depredated, one had either been depredated or hit by a mower, and one was found dead with no apparent injuries.

Six adults were soft-released and tracked for a total of 350 tracking days. Two of these individuals died, one was depredated by a Speckled Kingsnake (*Lampropeltis holbrooki*), and one was presumably depredated by a bird (it had been picked up and moved a long distance).

We tracked 47 resident juvenile lizards for a total of 1469 tracking days. Four of these resident juveniles died (one depredated by a Speckled Kingsnake, one killed by a predator and pulled into a burrow, one found deceased with no apparent injuries, and the other presumably depredated by a bird, as it was found hanging in vegetation).

We soft-released 17 juvenile lizards and tracked them for a total of 1210 tracking days. Two soft-released juveniles died during the study—one was found deceased with no visible injuries, and the other was hit by a mower.

**Figure 3:**
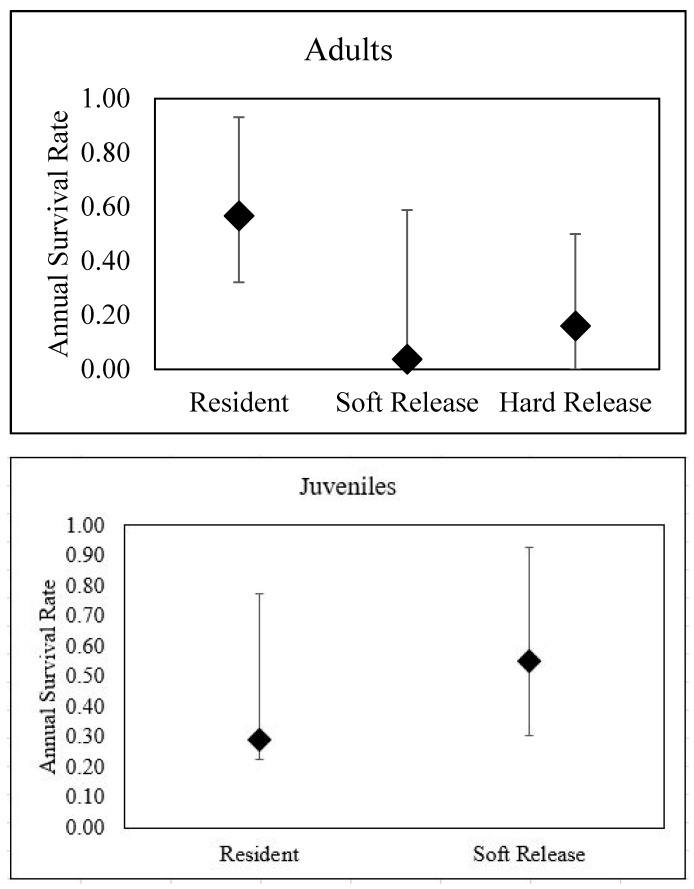
Estimated annual survival rates (±95% CI) for resident, soft-released, and hard-released adults (**top**) and juvenile (**bottom**) Texas horned lizards on Tinker Air Force Base, OK. Survival estimates for hard-released adult lizards come from 17 adult lizards translocated and tracked in 2008 [23].

For adult lizards, survival was most influenced by treatment (Table 2). Soft-released lizards had significantly lower estimated daily survival rates (0.998; 95% CI: 0.994–1.00) than residents (0.991; 95% CI: 0.972–0.997: Figure 3). Extrapolated to a 365 day annual cycle, survival rates for resident adult lizards were 57% (32–93%) and for soft-released lizards were 5% (1–59%). Treatment accounted for 81% of the weight of evidence, and was nearly 3 AICc units from the next competing model, which was day of year. Survival estimates for soft-released lizards overlapped extensively with the published estimates for hard-released lizards at this site (0.16; 95% CI: 0.00–0.34 [23]).

For juvenile lizards, both treatment and day of year were the top models, each accounting for approximately 50% of the weight of evidence, and were about 9 AICc units from the next competing models (Table 2). Soft-released juveniles had higher daily survival rates 0.998 (95% CI: 0.993–1.00) than resident juvenile lizards (0.997; 95% CI: 0.993–0.999), although there was considerable overlap in the confidence intervals (Figure 3). Juvenile lizard daily survival rates declined across the active season.

### 3.2. Movement

**Figure 4:**
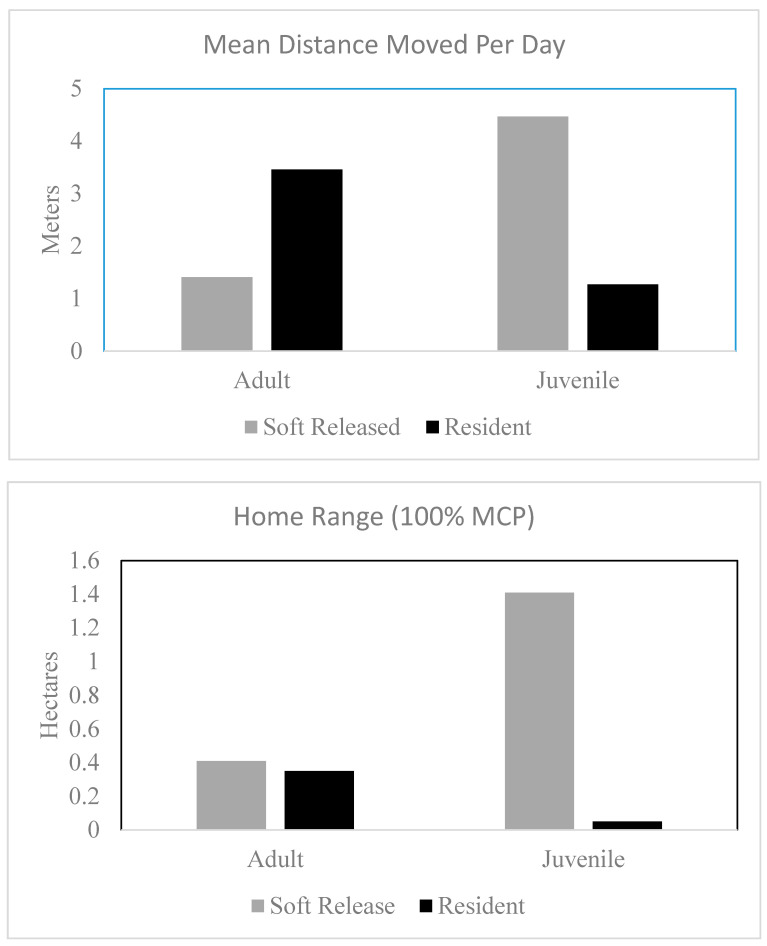
Average home range size (minimum convex polygons) and distance moved per day (+1 SD) of resident and translocated Texas horned lizards (*Phrynosoma cornutum*) on Tinker Air Force Base, OK.

Soft-released juveniles moved more per day than resident juveniles (χ^2^ = 10.21, df = 1, *p* = 0.001) and had larger overall home ranges (χ^2^ = 9.17, df = 1, *p* = 0.003). On average, soft-released juvenile lizards moved 4.5 m/day (±6.1 SD) and had MCP home ranges of 1.4 ha (±2.2 SD), whereas resident juveniles moved 1.3 m/day (±1.3 SD) and had MCP home ranges of 0.05 ha (Figure 4). There was no evidence that there was a difference in home range size between adult soft-released and resident lizards (χ^2^ = 0.17, df = 1, *p* = 0.68) or distance moved per day (χ^2^ = 1.75, df = 1, *p* = 0.19). Soft-released adult lizards moved on average 1.4 m/day (±0.5 SD) and had MCP home ranges of 0.4 ha (±0.4 SD), whereas resident adult lizards moved on average 3.5 m/day (±2.3 SD) and had MCP home ranges of 0.35 ha (±0.37 SD).

## 4. Discussion

The ultimate goal of wildlife translocation is to move individuals to a donor site where they can remain to augment or establish a population. Our results suggest that translocation has mixed utility for Texas horned lizard conservation. Adult lizards that experienced a 2-week soft release at our site had significantly lower survival rates than resident adults at the same site. Furthermore, these soft-released individuals had similar, low rates of survival as hard-released adult lizards from a previous study at this site [23]. Although the reported survival rates for adult Texas horned lizards are quite variable (9–59% [22,28]), the results from translocated adult lizards in this study are below what has previously been reported for the species, and are likely unsustainable. Given the low survival rates of translocated adult Texas horned lizards at this site, we urge managers and practitioners to use caution and explore alternatives before adopting translocation as a conservation tool for adults of this species.

Surprisingly, we found an age-dependent effect on the success of soft-release translocation for Texas Horned Lizards. Our results for soft-release survival of juveniles appeared to be very promising, with translocated lizards having similar survival rates to resident juvenile lizards. A previous study at this site hard-released adult lizards [23], but did not move any juvenile lizards, so we are unclear if juveniles at this site respond well to soft-release translocation or translocation in general. It remains to be seen how juvenile Texas horned lizards would respond to hard release translocation.

Translocation of juveniles is a frequently employed strategy in the fisheries world, where young, captive-reared fish are the typical release cohort, and several studies have found that these juveniles respond well to soft release, with either higher survival [29,30,31] or reduced dispersal [32]. However, we are aware of very few studies that explicitly compared the survival of soft-released adults and juveniles. One study found that soft-released subadult foxes (*Vulpes velox*) had higher short-term survival than did soft-released adults [33]. Campbell and Croft [34] showed that younger (<18 mo) soft-released kangaroos (*Macropus giganteus*) established home ranges that overlapped more with resident animals than did soft-released kangaroos of older age classes, suggesting that younger individuals more quickly integrated into the population. Further studies comparing the responses of juveniles or hatchlings to adults in soft-release translocation will be valuable and better inform practitioners. The approach certainly needs more examination, as some practitioners have suggested that neonates may be less suitable for translocations due to their propensity to disperse [35]. A significant limitation of our study is that we did not hard-release juveniles, so we do not know if juvenile lizards respond well to soft release in particular or translocation in general.

One of the mechanisms by which soft release should improve survival is by reducing post-release movement [36]. However, this does not appear to be the mechanism improving the survival of the translocated juvenile lizards. The soft-released juvenile lizards we tracked occupied larger home ranges and moved more per day than resident juveniles. Thus, it is unclear what the mechanism underlying the high survival rates was. Perhaps soft release simply reduces stress and provides the lizards an opportunity to settle down and recuperate resources while being protected from most predators during a time that they would be most vulnerable [36,37]. Alternatively, because horned lizards rely heavily upon crypsis [38], it may give them an acclimation period where they better adjust to their new physical surroundings.

Soft release has many examples of success (e.g., [13,14]), although there are numerous reports indicating that the technique had little effect on post-release behavior [39,40,41], and rare cases where it negatively impacted translocation success [42]. Relatively few published studies have used soft release in conjunction with the translocation of lizard species. However, Miller et al. [17] reported that soft-released adult Texas horned lizards had similar movement and survival rates to those reported in the literature for resident animals. Another study using soft release with jeweled geckos (*Naultinus gemmeus*) reported that soft release reduced post-release dispersal movements [15]. We found little evidence that soft release provides adult lizards with a survival benefit relative to hard release methods. We only kept lizards in pens for 2 weeks before release—it is possible that our chosen length of time was insufficient to prevent lizards from making large erratic movements and making themselves vulnerable to predators upon release. Knox and Monks [15] confined translocated lizards in their study for a period of 9–10 months. Similarly, longer holding times, sometimes up to years, has proven successful for other adult reptile species [13]. Future studies would benefit by experimentally examining penning duration to identify optimal confinement times.

Translocation is such a high-risk conservation strategy that any improvement to its success rate may have material benefits for species conservation. However, more studies comparing, demonstrating, and validating the effectiveness of translocation strategies is desperately needed. Our results have shown that moving juveniles may be a more viable strategy for soft-release translocation than moving adults. Future efforts should further investigate this phenomenon to explore how widespread it is.

## 5. Conclusions

Soft release is a promising technique for improving the success of wildlife translocation programs; however, further empirical research is needed. Our results suggest that soft release is not a viable approach for improving the survival of adult lizards on Tinker Air Force Base. However, our results did show that translocated juveniles had very high post-release survival. We need to understand if juveniles responded directly to the soft release approach, or if they are simply more resilient to translocation in general. Further research is needed to explore the underlying mechanism that leads to high juvenile survival after soft release, and whether alternative strategies exist to improve adult survival following translocation.

## Figures and Tables

**Table 1:** Number of resident and translocated Texas horned lizards (*Phyrynosa cornutum*) tracked on Tinker Air Force Base, OK, between 2016–2018.

Adults	2016	2017	2018
Residents	0	8	9
Soft-Released	2	1	3
**Juveniles**			
Residents	1	1	45
Soft-Released	3	10	4

**Table 2:** Comparison of four candidate models examining effect of variables on post-release survival of resident and translocated Texas horned lizards (*Phrynosoma cornutum*).

Model		Loglike	*K*	AIC	ΔAICc	wi
Adults	Treatment	−19.412	2	48.824	0.000	0.811
	Day of Year	−20.868	2	51.736	2.912	0.189
	Pen	−18.771	3	67.541	18.717	0.000
	Year	−19.249	3	68.497	19.673	0.000
Juveniles	Treatment	−30.487	2	70.974	0.000	0.499
	Day of Year	−30.494	2	70.988	0.014	0.496
	Year	−25.045	3	80.089	9.115	0.005
	Pen	−30.483	3	90.966	19.992	0.000

Loglike = log-likelihood, *K* = the number of parameters, AIC = Aikaike information criterion, ΔAICc = difference between AIC and the minimum AIC, wi = weight of evidence.
